# Population structure and regeneration constraints of *Magnolia zenii* (Magnoliaceae) in Baohua Mountain, Jiangsu, China: Insights for conservation strategies

**DOI:** 10.3897/BDJ.13.e175563

**Published:** 2025-11-17

**Authors:** Chunping Xie, Dawei Liu, Cheng Liang

**Affiliations:** 1 Tropical Biodiversity and Bioresource Utilization Laboratory, Qiongtai Normal University, Haikou, China Tropical Biodiversity and Bioresource Utilization Laboratory, Qiongtai Normal University Haikou China; 2 Faculty of Criminal Science & Technology, Nanjing Police University, Nanjing, China Faculty of Criminal Science & Technology, Nanjing Police University Nanjing China; 3 Environmental Resources and Food and Drug Crime Investigation Team, Yunnan Provincial Public Security Department, Kunming, China Environmental Resources and Food and Drug Crime Investigation Team, Yunnan Provincial Public Security Department Kunming China

**Keywords:** *Magnolia zenii*, extremely small population, population structure, size class, Baohua Mountain

## Abstract

**Background:**

*Magnolia zenii* Cheng, an endemic and critically endangered species, is restricted to Baohua Mountain, Jiangsu Province, China. Despite the implementation of in situ conservation measures implemented after the establishment of Baohua Mountain Nature Reserve in 1984, the species continues to face significant regeneration challenges.

**New information:**

This study evaluates the population structure and regeneration dynamics of *M. zenii* using data from field surveys conducted in 2001 and 2024. Our findings indicate a substantial increase in the total population size from 34 to 115 individuals and a broadening of the size-class distribution. However, persistent gaps in recruitment, particularly the absence of individuals in the smallest diameter class (< 5 cm), highlight critical reproductive and establishment constraints. Factors limiting regeneration include poor seedling establishment due to reproductive inefficiencies, intense competition from *Phoebe sheareri* and *Phyllostachys edulis* and anthropogenic impacts associated with tourism management. We propose a multi-faceted conservation strategy emphasising enhanced reproductive management, mitigation of competitive pressure and science-based tourism control to promote sustainable population recovery. This study provides a comprehensive framework for addressing regeneration constraints and providing information for long-term conservation policies for *M. zenii* and other endangered species with similar ecological challenges.

## Introduction

**Figure 1.**
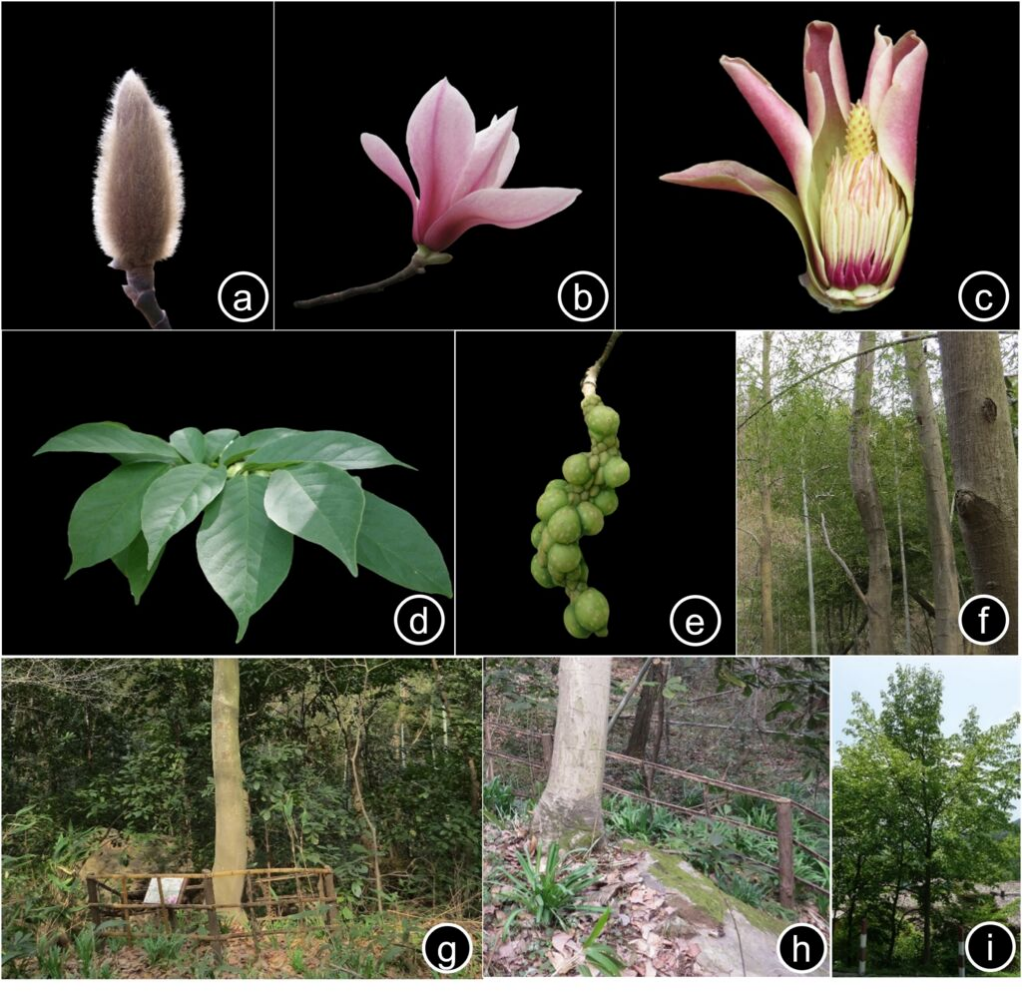
*Magnolia zenii*: **a** bud; **b** blooming flower; **c** exposed stamens and pistils; **d** leaves; **e** aggregate follicles; **f** community co-existing with *Phyllostachys edulis*; **g** garden plants decorating around the target tree; **h** cement-hardened ground ; **i** outline of the cultivated tree along the tourist road.

The Magnoliaceae, an ancient and critical angiosperm family (Doyle and Endress 2023), exhibits a predominantly tropical and subtropical distribution, with peak abundance observed within 10° of latitude of the Tropics of Cancer and Capricorn (Zhu et al. 2016, Ashton and Zhu 2020, Shankar 2020). Globally, the family comprises approximately 300 species (Wang et al. 2020, Linsky et al. 2023), with China representing a centre of diversity, harbouring more than 160 species (Xie et al. 2022). Within Chinese subtropical forest ecosystems, the Magnoliaceae, alongside the Lauraceae, Fagaceae and Theaceae, contribute significantly to ecosystem function, maintaining regional ecological balance and supporting biodiversity (Zhu et al. 2016, Xie et al. 2022). These plants possess diverse applications, notably in horticulture (Fig. 1b and i), traditional medicine and as sources of spices (Mir et al. 2020). However, anthropogenic pressures, including habitat destruction, coupled with inherent reproductive limitations, have resulted in significant population declines, with numerous species categorised as vulnerable, endangered or critically endangered (Hernández et al. 2020, Linsky et al. 2023). Consequently, 24 Magnoliaceae species are designated as nationally protected wild plants in China (National Forestry and Grassland Administration and Ministry of Agriculture and Rural Affairs 2021), such as *Magnolia zenii*, *Liriodendron chinense*, *Houpoea officinalis*, *Michelia shiluensis*, *Pachylarnax sinica* and *Woonyoungia septentrionalis*.

*Magnolia zenii* Cheng (1933) is endemic to China and is considered a species with an extremely small population size and narrow distribution (Wang et al. 2024). It is classified as critically endangered (CR) on the IUCN Red List of Threatened Species (Workshop 2014). The species was first discovered and described by the Chinese botanist Cheng Wan-Chun in 1933 (Fig. 1a-e) (Type specimen No. 4233, Herbarium of the Institute of Botany, Chinese Academy of Sciences, PE) (Hao et al. 2000), within the secondary forest of Baohua Mountain in Jiangsu Province. This discovery has been of significant value to the taxonomic study of the Magnoliaceae. Due to its specific environmental requirements, the seedling establishment rate of *M. zenii* under natural conditions is low (Jiang et al. 2010), contributing to its extremely limited population and imminent risk of extinction. Historically, the wild population of *M. zenii* has been documented exclusively on Baohua Mountain, Jiangsu Province (Hao et al. 2000). To date, this single population remains critically scarce, underscoring the urgent need for conservation efforts to ensure its long-term survival.

This study is based on field surveys and population data from the 2001 and 2024 surveys. It aims to: (1) compare and analyse the population dynamic since the establishment of the Nature Reserve and (2) explore the challenges hindering its growth, thereby providing valuable scientific insights for the conservation of this endangered species.

## Study Area

**Figure 2.**
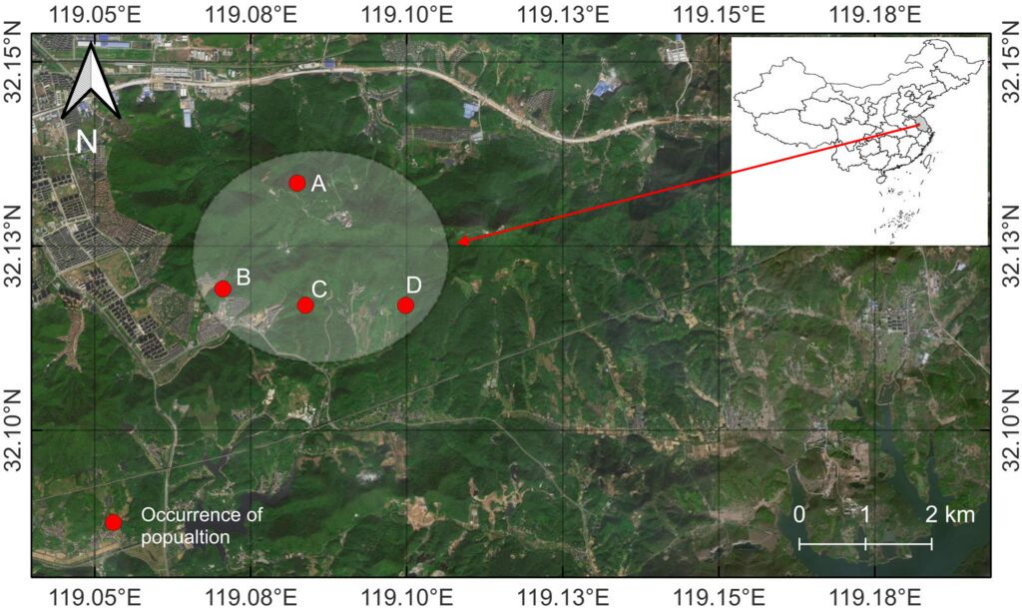
Location of the population of *Magnolia zenii* on Baohua Mountain, Jiangsu, China. The endangered population is highly concentrated in just four distinct zones of Baohua Mountain, with these areas separated by no more than 2 km from each other. The mountain is heavily encroached upon by human development, including extensive residential complexes, road networks and railway infrastructure. This intense human activity has severely impacted the local population, while simultaneously fragmenting their natural habitat into increasingly isolated patches.

The study was conducted in Baohua Mountain National Nature Reserve (119°02′-119°08′E, 32°05′-32°09′N), located in Jurong, Jiangsu Province, eastern China. Baohua Mountain covers an area of approximately 2,000 hectares, with elevations ranging from 100 to 445 m above sea level. The region experiences a subtropical monsoon climate, characterised by an average annual temperature of 15.6°C and annual precipitation of about 1,100 mm. The vegetation is dominated by subtropical evergreen broad-leaved forests, with key species including *Phoebe sheareri*, *Phyllostachys edulis* and other members of the Magnoliaceae, Lauraceae and Fagaceae families. *M. zenii* is endemic to this area and is primarily distributed in four fragmented zones within the Reserve, separated by distances of no more than 2 km, amid increasing anthropogenic disturbances, such as tourism infrastructure, residential areas and habitat fragmentation (Fig. 2).

## Materials and Methods

Population data for *M. zenii* were obtained from two comprehensive field surveys conducted in 2001 and 2024. The 2001 survey, as documented by Xu et al. (2001), involved systematic censuses of all known individuals in the study areas of the Reserve. In 2024, a follow-up survey was performed by Li et al. (2024), employing similar protocols to ensure comparability. Both surveys targeted the entire known population across the four distribution zones. Individuals were categorised into 10 DBH (diameter at breast height) size classes for analysis: Class I (0–5 cm), II (5–10 cm), III (10–15 cm), IV (15–20 cm), V (20–25 cm), VI (25–30 cm), VII (30–35 cm), VIII (35–40 cm), IX (40–45 cm) and X (≥ 45 cm). This classification allowed for the evaluation of population structure and regeneration dynamics, following standard methods for tree population studies (Brzeziecki et al. 2016). Frequency distributions of individuals across size classes were calculated for both the 2001 and 2024 datasets to visualise changes in population structure. Population growth was quantified as the absolute increase in total individuals and the proportional changes within each size class. Gaps in recruitment were identified by the absence or low frequency of individuals in the smaller size classes (< 5 cm DBH). To quantitatively compare the population structures between 2001 and 2024, a *t*-test was applied to the frequency distributions across the 10 DBH size classes, confirming significant differences in mid-size class expansions (*p* < 0.05).

To assess interspecific interactions, vegetation plots (20 m × 20 m) were established around clusters of *M. zenii* individuals. These plots were centred on the densest clusters of *M. zenii* within each of the four distribution zones, selected to represent typical community composition while minimising overlap with heavily disturbed areas. Within these plots, dominant species (e.g. *P. sheareri* and *P. edulis*) were identified and their density, DBH and coverage were recorded. Anthropogenic impacts were evaluated by documenting tourism-related modifications, such as hardened paths, landscaping and visitor traffic in proximity to *M. zenii* habitats. No destructive sampling was conducted to minimise the impact on this critically endangered species. All field activities adhered to the guidelines of the Baohua Mountain Nature Reserve management authority. During the surveys, plots were located and mapped using handheld GPS devices for precise georeferencing within the distribution zones. DBH was measured using standard diameter tapes or digital calipers to ensure accuracy, while tree heights were estimated using clinometers or laser rangefinders as needed for taller specimens.

## Results and Discussion

Prior to the establishment of the Baohua Mountain Nature Reserve in 1984, the known *M. zenii* population consisted of approximately a dozen mature trees, with the largest individual measuring 30 cm in DBH and 14 m in height (Hao et al. 2000). A survey conducted in 1999 recorded 34 adult individuals of *M. zenii* on Baohua Mountain, primarily concentrated in the Reserve's low-lying areas (Xu et al. 2001). Subsequent studies of the population and its associated community were conducted in 2001 (Xu et al. 2001), 2008 (Wang et al. 2008), 2019 (Wang et al. 2019) and 2024 (Li et al. 2024).

**Figure 3.**
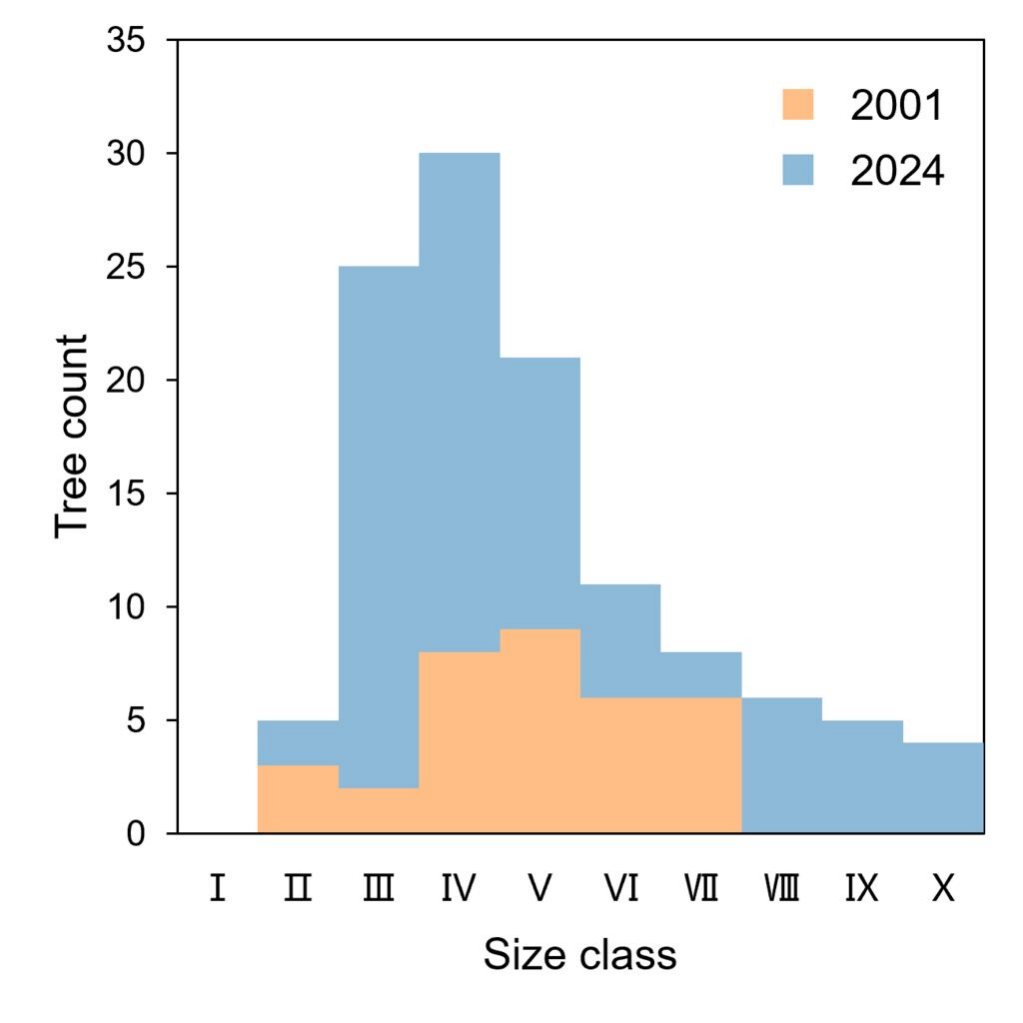
Frequency distribution of tree counts for *Magnolia zenii* populations on Baohua Mountain, Jiangsu, China, in 2001 and 2024. Size class from Ⅰ to Ⅹ denote 0 – 5 cm, 5 – 10 cm, ... and ≥ 45 cm.

Our analysis of *M. zenii* population dynamics from 2001 (Xu et al. 2001) to 2024 (Li et al. 2024) revealed substantial structural changes and a marked increase in the total population size from 34 to 115 individuals (Fig. 3). A *t*-test applied to the frequency distributions across the 10 DBH size classes confirmed significant differences in population structure between the two survey years (*t* = 2.37, *p* = 0.029). While the population showed significant expansion in the middle diameter classes (10-20 cm) and the emergence of large individuals (> 35 cm) previously absent in 2001, we identified critical gaps in regeneration, evidenced by the persistent absence of individuals in the smallest diameter class (0-5 cm). The population structure shifted from a concentrated distribution in the 15-30 cm diameter classes in 2001 to a broader, but uneven distribution across size classes by 2024, with the most pronounced increase occurring in the 10-20 cm diameter classes (from 10 to 55 individuals). This growth can be attributed to natural expansion over the 23-year interval, supported by improved protection measures in the Reserve that enhanced survival and maturation rates of existing trees. Although the overall population growth suggests improved survival rates of established individuals, the lack of small-diameter trees raises concerns about recruitment limitations and long-term population sustainability (Brzeziecki et al. 2016), indicating potential barriers to natural regeneration that warrant further investigation and targeted conservation measures.

Despite notable success in population enhancement through in situ conservation efforts, the demographic structure of *M. zenii* reveals persistent challenges in recruitment, particularly evidenced by the absence of individuals in the < 5 cm diameter class (Fig. 3). Our investigation identifies three primary limiting factors affecting population regeneration: firstly, the reproductive biology of *M. zenii* presents significant constraints to natural regeneration. Upon maturity, the species' aggregate fruits fall to the ground, where seeds typically undergo decay within the fruit structure, severely compromising germination potential (Wang et al. 2018); similar situations have also been found in other plants of the Magnoliaceae (Galván-Hernández et al. 2020). Field observations confirm this reproductive limitation, with notably scarce seedling presence in natural forest stands. While sexual maturity is reached at 5-7 years, successful reproduction is further hindered by low fruit set rates and marked temporal variation in reproductive success. In particular, fruit production shows a significant negative correlation with drought conditions, exhibiting substantial inter-annual fluctuations (Zhang 2007). These biological characteristics, coupled with environmental sensitivities, create substantial obstacles to natural regeneration, contributing to the species' limited recruitment success.

Interspecific competition significantly impacts the recruitment and establishment of *M. zenii* in its native habitat on Baohua Mountain (Jiang et al. 2010), where it exists as a companion species within communities dominated by *P. sheareri* and *P. edulis* (Fig. 1f and g). These dominant species exhibit superior ecological adaptations, including robust germination capabilities, shade tolerance and drought resistance, enabling them to thrive in the steep, rocky terrain (Wang et al. 2019). While mature *M. zenii* can access deep water and nutrient resources through their extensive taproot systems, seedling establishment is severely compromised by multiple competitive pressures. The dense canopy created by *P. sheareri* limits light penetration to the forest floor (Zhang et al. 2019), particularly affecting *M. zenii* seedlings due to their decreasing shade tolerance during development (Wang et al. 2018). Furthermore, the extensive rhizome network of *P. edulis* creates intense below-ground competition for soil resources (Yu et al. 2024), effectively suppressing seedling and sapling development. This combination of above -and below-ground competitive interactions creates significant barriers to the natural regeneration of *M. zenii* within its native community structure.

Thirdly, tourism development and associated management practices in the Baohua Mountain Nature Reserve have created unintended negative consequences for *M. zenii* conservation (Fig. 1g and h) despite the species' flagship status. While landscape beautification initiatives around mature *M. zenii* individuals were implemented to enhance the visitor experience, these activities have inadvertently compromised natural regeneration processes through the direct removal of seedlings during landscaping operations. Given that *M. zenii* relies primarily on natural regeneration for population maintenance, such habitat modifications significantly impede population recovery and expansion. Furthermore, increased tourist activity has intensified anthropogenic pressures on sensitive regeneration zones, resulting in soil compaction and physical damage to seedlings through trampling (Kalahroudi et al. 2023). Although protective measures exist in designated areas, inadequate enforcement of management protocols has failed to mitigate these impacts effectively. The confluence of these factors - landscape modification, tourism pressure and insufficient science-based management strategies - has exacerbated the challenges facing *M. zenii* recruitment, highlighting the conflict between tourism development and species conservation objectives.

## Implications and conclusion

A comprehensive conservation strategy for *M. zenii* requires an integrated approach addressing multiple challenges: (1) To enhance reproductive success and seedling establishment, we propose implementing specialised seed handling protocols, establishing nursery programmes and optimising germination conditions. For instance, a straightforward reproductive management step could involve manual seed collection from mature aggregate fruits during peak ripeness (typically late summer), followed by immediate scarification and stratification in a controlled moist environment at 4–10°C for 3–6 months to break dormancy and improve germination rates, before transplanting viable seedlings to protected nursery beds within the Reserve; (2) Competitive pressure management should focus on selective canopy modification of *P. sheareri* and rhizome control of *P. edulis* to create favourable light conditions and reduce below-ground competition; (3) Tourism impact mitigation requires infrastructure modifications, including elevated walkways and clear physical barriers, complemented by science-based landscaping protocols that prioritise seedling protection. These efforts should be supported by robust research and monitoring programmes to track population demographics, study microhabitat requirements and assess genetic diversity. The success of these initiatives depends on effective stakeholder engagement through staff training, public education and community involvement in conservation efforts. Finally, the implementation of clear management policies that balance conservation objectives with tourism development, supported by secure long-term funding and regular strategy review, is essential for the species' long-term survival.

## Species Conservation Profiles

### Magnolia zenii

#### Species information

Scientific name: Magnolia zenii

Species authority: Cheng

Synonyms:

*Yulania zenii* (W.C.Cheng) D.L.Fu

*Magnolia elliptilimba* Y.W.Law & Z.Y.Gao

Common names: Baohua Yulan

Kingdom: Plantae

Phylum: Tracheophyta

Class: Magnoliopsida

Order: Magnoliales

Family: Magnoliaceae

Figure(s) or Photo(s): Fig. 1

Region for assessment: Global

#### Editor & Reviewers

##### Reviewers

Reviewers: Chunping Xie，Dawei Liu

##### Editor

Editor: Chunping Xie

#### Geographic range

Biogeographic realm: Palearctic

Countries: China

Map of records (image): Fig. 2

Map of records (Google Earth): none

Basis of EOO and AOO: Unknown

Basis (narrative): Unknown

Min Elevation/Depth (m): 200

Max Elevation/Depth (m): 220

Range description: Endemic species of China, only recorded in Baohua Moutain, Jiangsu Provice.

#### Extent of occurrence

EOO (km2): 107200

Trend: Unknown

Causes ceased?: Unknown

Causes understood?: Unknown

Causes reversible?: Unknown

Extreme fluctuations?: Unknown

#### Area of occupancy

Trend: Unknown

Causes ceased?: Unknown

Causes understood?: Unknown

Causes reversible?: Unknown

Extreme fluctuations?: Unknown

AOO (km2): 140

#### Locations

Number of locations: 1

Justification for number of locations: The species is documented in one location distributed in Baohua Mountain, Jiangsu Province.

Trend: Unknown

Extreme fluctuations?: No

#### Population

Trend: Unknown

Causes ceased?: Unknown

Causes understood?: Unknown

Causes reversible?: Unknown

Extreme fluctuations?: Yes

#### Subpopulations

Trend: Unknown

Extreme fluctuations?: Yes

Severe fragmentation?: Yes

#### Habitat

System: Terrestrial

Habitat specialist: No

Habitat (narrative): It survives in the evergreen-deciduous broadleaf mixed forest and deciduous broadleaf forest on Baohua Mountain, Jiangsu Province.

Trend in extent, area or quality?: Unknown

##### Habitat

Habitat importance: Major Importance

Habitats: 1. Forest

#### Ecology

Size: Unknown

Generation length (yr): 0

Dependency of single sp?: Unknown

Dependent on IUCN Status: Critically Endangered (CR)

Ecology and traits (narrative): Terrestrial

#### Threats

##### Threats

Threat type: Ongoing

Threats: 1. Residential & commercial development4. Transportation & service corridors6. Human intrusions & disturbance

#### Conservation

##### Conservation actions

Conservation action type: In Place

Conservation actions: 3.2. Species management - Species recovery3.3. Species management - Species re-introduction

#### Other

##### Use and trade

Use type: International

Use and trade: 6. Other chemicals14. Research

##### Ecosystem services

Ecosystem service type: Very important
